# Multi-Omics Integration Drives Precision Breeding in Sorghum: Molecular Dissection and Breeding Practice

**DOI:** 10.3390/plants15192954

**Published:** 2026-09-28

**Authors:** Wenfang Zhou, Xiaoyan Chen, Yuntong Lu, Fei Li

**Affiliations:** School of Life Sciences, Guizhou Normal University, Guiyang 550001, China; 252100100478@gznu.edu.cn (W.Z.);

**Keywords:** sorghum, multi-omics integration, precision breeding, molecular dissection, gene regulatory network, stress tolerance

## Abstract

Sorghum (*Sorghum bicolor* L.), the fifth-most important cereal crop globally, serves as a cornerstone crop for food security, forage production, and bioenergy feedstock on arid and semi-arid marginal lands, owing to its high water use efficiency derived from C4 photosynthesis and exceptional tolerance to abiotic stresses. Conventional sorghum breeding has long been constrained by insufficient genetic dissection of complex traits, the “black box” of genotype-to-phenotype mapping, and poorly understood genotype-by-environment interactions, resulting in stagnant genetic gain. In recent years, rapid advances in multi-omics technologies—including genomics, transcriptomics, epigenomics, single-cell omics, and microbiomics—have propelled sorghum research from single-gene mapping to systems-level dissection of regulatory networks, providing a novel paradigm for elucidating the molecular basis of agronomic traits across all molecular layers and breaking through the bottlenecks of conventional breeding. This review systematically synthesizes recent advances in sorghum multi-omics research, dissecting the molecular basis of key agronomic traits across distinct omics layers. We summarize the integrated multi-omics precision breeding technology system and elaborate on the practical applications of multi-omics approaches for four core breeding objectives: stress tolerance, yield, quality, and nutrient use efficiency. Finally, we discuss current challenges and future perspectives, aiming to provide a theoretical framework and technical reference for molecular design breeding in sorghum.

## 1. Introduction

Domesticated in Africa approximately 8000 years ago, sorghum is currently cultivated in more than 100 countries and regions worldwide, serving as a staple food for over 500 million people, while also possessing diversified industrial value as forage, brewing feedstock, and bioenergy material [1,2,3]. As a typical C4 crop, sorghum exhibits higher photosynthetic efficiency, water use efficiency, and nutrient use efficiency than C3 crops, with strong adaptation to drought, salinity, low soil fertility, and high temperature, making it an ideal crop for climate change adaptation, marginal land development, and ensuring food security [4,5]. China is the world’s largest sorghum importer, with a unique industrial structure: over 80% of sorghum consumption is used for Baijiu (Chinese liquor) production, while the planting area of forage sorghum and sweet sorghum continues to expand, creating an urgent demand for high-yield, high-quality, multi-resistant, and end-use-specific sorghum varieties [6].

Conventional sorghum breeding has progressed through phases of domestication, phenotypic selection, and heterosis utilization, making critical contributions to varietal improvement over the past decades. However, as breeding objectives become more diversified and complex, the bottlenecks of conventional approaches have become increasingly prominent: First, the most important agronomic traits (e.g., stress tolerance, quality, heterosis) are quantitative traits controlled by multiple genes with complex genetic architecture, leading to low efficiency and long cycles of phenotypic selection. Second, a single reference genome cannot capture all genetic variation within the species; a large number of structural variants and non-coding variants associated with important traits are overlooked, which constitutes one important source of the “missing heritability” problem. Third, genotype-by-environment interactions remain poorly understood, making it difficult to predict varietal environmental adaptability, requiring breeders to conduct multi-year, multi-location evaluations across environments at extremely high cost [7,8,9].

Since the beginning of the 21st century, revolutionary breakthroughs in high-throughput sequencing technologies have driven rapid advances in crop omics research. From reference genome construction to pan-genome analysis, from bulk transcriptomics to single-cell resolution gene expression atlases, from host genomics to host–microbiome co-analysis, multi-omics technologies have systematically dissected the complete regulatory chain from genotype to phenotype across distinct molecular layers, providing novel solutions to overcome conventional breeding bottlenecks [10]. In sorghum research, multi-omics technologies have not only enabled rapid cloning of important functional genes, but also revealed the regulatory networks underlying trait formation and environmental response mechanisms, driving the transition from “experience-based breeding” to “precision design breeding” [11].

Existing reviews on sorghum omics have largely focused on individual omics technologies, molecular mechanisms of single trait classes, or standalone breeding technology updates. Systematic integration of layered multi-omics technologies and the full translational pathway from mechanistic dissection to breeding application remains less thoroughly addressed. Following the central thread of “genetic variation dissection—regulatory network construction—breeding technology integration—trait improvement practice”, this review systematically integrates multi-dimensional research findings from genomics, transcriptomics, epigenomics, single-cell omics, and microbiomics. We discuss how multi-omics technologies dissect the molecular basis of key sorghum agronomic traits, how they integrate to form a precision breeding technology system, and their practical applications in core breeding objectives. Finally, we discuss future directions for multi-omics-driven intelligent design breeding in sorghum. This review distinguishes itself by constructing a complete logical framework from molecular dissection of key agronomic traits to integrated precision breeding systems, to practical breeding deployment. It further addresses industry-specific breeding demands and translational bottlenecks, providing an actionable reference for sorghum molecular breeding practice.

## 2. Molecular Basis of Sorghum Traits Dissected by Layered Multi-Omics

The formation of agronomic traits is a multi-layered regulatory process ranging from genomic variation to transcriptional regulation, epigenetic modification, cell differentiation, individual development, and ultimately interactions with the environment and microbiota. Distinct omics technologies dissect the molecular mechanisms of this process from different molecular levels, collectively constituting the complementary multi-layered insights that substantially advance trait dissection (Figure 1).

### 2.1. Genomics: Panoramic Germplasm Variation and the Genetic Foundation of Domestication and Improvement

The genome serves as the genetic basis for all traits. Sorghum genomics research has evolved from a single reference genome to pan-genomes, and from single nucleotide variants to full-scale structural variants, providing critical insights into trait regulatory mechanisms. The release of the first sorghum reference genome BTx623 v1.0 in 2009 marked the beginning of the genomic era for sorghum. Over the subsequent decade, genomes of multiple inbred lines including RTx430, Rio, and Ji2731 were successively assembled, laying the foundation for gene mapping and comparative genomics research [11,12,13]. In recent years, advances in PacBio HiFi, ONT long-read, and Hi-C technologies have propelled sorghum genome assembly into the telomere-to-telomere (T2T) complete genome era. Multiple T2T genomes, including BTx623, Chinese inbred line Ji2055, and Baijiu-brewing landraces Hongyingzi and Huanjie nuo, have been released, filling assembly gaps in complex repetitive regions such as centromeres and telomeres, correcting assembly errors in previous versions, and providing high-quality references for accurate dissection of gene structure and complex variants [14,15,16].

A single reference genome cannot capture all genetic variation within a species. Pan-genome studies, through de novo assembly of multiple representative germplasms, have constructed the complete gene set of sorghum. Currently released sorghum pan-genomes cover hundreds of accessions ranging from wild relatives to different types of cultivated sorghum, identifying approximately 30,000 core genes (present in all accessions) and a large number of variable genes (present only in a subset of accessions). Variable genes are frequently associated with biotic and abiotic stress responses, representing an important genetic basis for sorghum environmental adaptability [17,18]. Pan-genome studies have not only revealed selection signatures during sorghum domestication, such as selection footprints at key loci including the seed shattering gene *Sh1*, juicy stem gene *Dry*, and tannin gene *Tan1*, but also identified numerous favorable allelic variants missed by the reference genome, providing abundant genetic resources for breeding [19,20].

For a long time, genomic research primarily focused on single-nucleotide polymorphisms (SNPs) and small insertions/deletions (InDels). However, accumulating evidence demonstrates that structural variants (SVs), including large deletions, insertions, duplications, inversions, and translocations, make substantial contributions to phenotypic variation and environmental adaptation. Whole-genome SV analysis of 363 global sorghum accessions identified over 620,000 high-quality SVs, revealing that SVs are enriched in gene-rich regions and exhibit low linkage disequilibrium with surrounding SNPs, and thus cannot be effectively tagged by SNP markers. Incorporating SVs into genome-wide association studies (GWAS) enabled identification of numerous trait-associated loci undetectable by SNPs alone, significantly increasing the explained heritability for traits including flowering time, plant height, and biomass, and revealing genomic hotspots underlying differentiation between cellulosic and sweet sorghum [21]. Furthermore, complex structural variants such as copy number variations (CNVs) and presence/absence variations (PAVs) have also been shown to be closely associated with sorghum stress tolerance and quality traits; for example, functional variants at the striga resistance locus *LGS1* and seed shattering gene *Sh1* are both caused by complex structural variants [22,23].

Non-coding region variation represents an important source of “missing heritability”. Over 80% of the sorghum genome consists of non-coding regions, containing a large number of cis-regulatory elements such as promoters, enhancers, and silencers. Variation in these regions can affect phenotypes by altering gene expression levels and spatiotemporal expression patterns. For example, the dw3 mutant allele carries an 882 bp tandem duplication within the fifth exon of the gene, resulting in the dwarf phenotype; variation in the *Tan1* promoter region leads to downregulation of tannin biosynthesis pathway genes, producing tannin-free grains [24,25]. With the application of assays for transposase-accessible chromatin sequencing (ATAC-seq) and chromatin immunoprecipitation sequencing (ChIP-seq), the cis-regulatory element landscape of sorghum is being systematically dissected, providing a foundation for mining breeding targets in non-coding regions. As the foundational layer of heritable variation, genomic variants provide the primary genetic basis for phenotypic differences; their functional interpretation requires integration with transcriptomic and epigenomic evidence to link sequence changes to trait expression.

### 2.2. Transcriptome–Epigenome Coordination: Molecular Networks Underlying Dynamic Trait Regulation

While the genome provides the genetic potential for traits, spatiotemporally specific gene expression determines the dynamic process of trait formation. Transcriptomics and epigenomics dissect the molecular networks underlying trait development and environmental responses at the level of gene expression regulation. RNA sequencing (RNA-seq) has been widely applied to profile gene expression across different sorghum tissues, developmental stages, and stress treatments, constructing a spatiotemporal transcriptome atlas for sorghum. In developmental regulation, transcriptomic studies have revealed core regulatory pathways for important traits including flowering time, plant height, and panicle development: the jasmonic acid biosynthesis pathway determines grain number per panicle by regulating fertility of pedicellate spikelets; brassinosteroid and auxin signaling pathways determine plant height by regulating internode cell elongation; and the circadian clock–photoperiod pathway regulates flowering time by integrating light signals [26,27,28]. In stress responses, transcriptomic studies have systematically dissected gene expression reprogramming in sorghum under drought, salinity, high temperature, and pathogen challenges: Integrated transcriptomic and metabolomic analysis of two sorghum genotypes with contrasting heat tolerance revealed that heat-tolerant genotypes constitutively overexpress heat shock protein (HSP) and heat shock transcription factor (HSF) genes under heat stress, while simultaneously inducing upregulation of genes in the raffinose family oligosaccharide (RFO) biosynthesis pathway and reactive oxygen species (ROS) scavenging pathway, and repressing the SnRK1 energy sensing pathway, achieving thermotolerance through osmoprotection, protein stabilization, and redox homeostasis maintenance [29]. Dual RNA-seq analysis of the interaction between sorghum and Colletotrichum sublineola simultaneously profiled transcriptome dynamics of both host and pathogen, revealing the “arms race” between host NLR immune receptor activation and pathogen effector expression, identifying multiple key sorghum disease resistance genes and pathogen pathogenicity factors [30].

Epigenetic modifications are important mechanisms regulating gene expression without altering DNA sequence, including DNA methylation, histone modifications, RNA methylation (m6A), and non-coding RNA regulation, playing critical roles in sorghum development, stress responses, and heterosis formation. Regarding DNA methylation, studies have shown that sorghum undergoes genome-wide alterations in methylation levels under drought and salt stress; demethylation of promoter regions of some stress-responsive genes leads to gene activation, and some stress-induced DNA methylation alterations can be stably propagated via mitotic cell divisions within the same individual, establishing somatic (intragenerational) stress memory, which improves stress responses during subsequent stress exposure throughout the plant life cycle. Regarding DNA methylation, studies have shown that drought and salt stress induce genome-wide reprogramming of methylation levels in sorghum. Demethylation of promoter regions of some stress-responsive genes leads to gene activation, and such stress-induced DNA methylation alterations can be stably propagated through mitotic cell divisions, establishing somatic (intragenerational) stress memory that improves stress responses throughout the plant life cycle. For such epigenetic marks to be passed onto progeny, modified cells must enter the reproductive lineage and escape epigenetic resetting during meiosis, giving rise to intergenerational or transgenerational stress memory. Direct experimental evidence for meiotically heritable epigenetic stress marks in sorghum remains limited, which constitutes one of the core bottlenecks for the breeding application of epigenetic traits [31,32].

For histone modifications, 19 histone deacetylase genes have been identified in sorghum, among which *SbHDT701* activates expression of biotic and abiotic stress-responsive genes by increasing histone acetylation levels at target genes; activating histone marks such as H3K4me3 and H3K36me3 are associated with active gene expression, while repressive marks such as H3K9me2 and H3K27me3 are associated with gene silencing. High expression of the disease resistance gene *ARG1* is directly associated with increased H3K4 and H3K36 methylation levels in its promoter region [33,34,35]. For RNA m6A methylation, expression of the sorghum m6A methyltransferase gene *SbMTA* is altered under salt stress, leading to changes in m6A modification levels and transcript abundance of ROS regulatory genes, affecting salt stress tolerance [32,36]. Additionally, long non-coding RNAs (lncRNAs) have also been shown to play regulatory roles in sorghum salt and drought stress responses, regulating expression of neighboring genes through cis- or trans-acting mechanisms. Stress-induced epigenetic marks differ substantially in stability and breeding utility. Promoter DNA methylation alterations are relatively stable across mitotic divisions, supporting somatic stress memory fixation within a single growing season. Most histone modifications, by contrast, are highly dynamic and readily reversed after stress relief. Notably, meiotically heritable epigenetic stress marks remain poorly documented in sorghum, consistent with the low transgenerational heritability bottleneck discussed in Section 5.

Integrated transcriptome and epigenome analysis provides a powerful tool for dissecting gene expression regulatory networks. Through multi-omics integrative analysis including expression quantitative trait loci (eQTL), methylation quantitative trait loci (mQTL), and ChIP-seq, cis-regulatory elements and trans-acting factors regulating gene expression can be systematically identified, constructing a complete regulatory chain from genetic variation to gene expression to phenotype, providing a wealth of regulatory region targets for breeding beyond coding regions. Transcriptomic data bridges genomic sequence variation and phenotypic output by capturing gene expression dynamics, and when paired with epigenomic profiles, further reveals the regulatory mechanisms underlying genotype-to-phenotype translation.

### 2.3. Single-Cell Omics: Developmental and Stress Response Mechanisms at Cellular Resolution

Conventional bulk RNA-seq sequences mixed cells from entire tissues or organs, which cannot capture intercellular heterogeneity and masks gene expression signatures of rare cell populations. The emergence of single-cell RNA sequencing (scRNA-seq) and spatial transcriptomics technologies has elevated the resolution of sorghum gene expression profiling to the single-cell level, providing a novel perspective for dissecting the cellular differentiation mechanisms underlying trait formation. Although sorghum single-cell omics research is still in its infancy, important progress has been made. Comparative single-cell transcriptome analysis of roots from three *Poaceae* species—maize, sorghum, and *Setaria viridis*—constructed a root cell atlas, identified conserved cell type regulatory modules and species-specific cell functional differentiation, and revealed molecular features of root cell evolution in C4 plants [37]. Spatial transcriptomics analysis of bioenergy sorghum stems mapped spatial expression profiles across different stem cell types, dissected gene expression signatures of vascular bundles, parenchyma, and sclerenchyma cell populations, and identified cell-type-specific regulators controlling secondary cell wall biosynthesis and sugar accumulation, providing targets for stem trait improvement in bioenergy sorghum [38].

Single-cell omics technologies hold broad application prospects in sorghum research: In developmental biology, they can dissect cell differentiation trajectories during key agronomic developmental processes including panicle differentiation, stomatal development, and vascular bundle formation, identifying key regulators of cell fate determination. In stress responses, they can reveal specific response mechanisms of key stress-responsive cell populations such as stomatal guard cells and root cortical cells, discovering stress tolerance functions of rare cell populations masked by bulk sequencing. In C4 photosynthesis research, they can dissect differentiation mechanisms of mesophyll cells and bundle sheath cells, as well as cell-type-specific regulatory networks of the C4 photosynthetic cycle, providing references for C4 rice engineering. With the development of single-cell multi-omics technologies (simultaneously profiling transcriptome, epigenome, and chromatin accessibility in single cells), future studies will systematically dissect sorghum gene regulatory networks at single-cell resolution, advancing understanding of trait formation mechanisms into the era of cellular precision. Epigenomic modifications add a regulatory layer that modulates transcriptional output independent of sequence changes, complementing genomic and transcriptomic evidence to explain previously unaccounted phenotypic variation.

### 2.4. Microbiome: A New Dimension of Trait Enhancement via Host–Microbiota Interactions

Plants are not isolated individuals, but form “holobionts” together with a large number of symbiotic microorganisms. The microbiome, often referred to as the plant’s “second genome”, can significantly affect crop phenotypes by regulating nutrient acquisition, stress tolerance, and immune responses, representing an important dimension that cannot be ignored for sorghum trait improvement. Sorghum microbiome research primarily focuses on rhizosphere and endophytic microbiomes. Studies have shown that sorghum microbiome assembly is co-regulated by host genotype, developmental stage, soil environment, and stress conditions. Under well-watered conditions, endophytic microbiome composition does not differ significantly between sorghum genotypes with contrasting yield performance. However, under drought stress, high-yielding sorghum undergoes significant and directional remodeling of root endophytic bacterial and fungal communities. This remodeling is primarily driven by host–microbe interactions rather than microbe–microbe interactions, indicating that high-yielding genotypes possess the genetic capacity to actively recruit beneficial microorganisms under stress [39]. Significant differences in microbiome composition exist among sorghum genotypes, demonstrating that microbiome recruitment is under host genetic control, providing a genetic basis for improving the microbiome through breeding [40].

Functional microorganisms can enhance sorghum stress tolerance and nutrient use efficiency through multiple mechanisms: For stress tolerance, *Actinobacteria* and *Firmicutes* enriched in the sorghum rhizosphere under drought stress promote root growth, maintain water balance, and improve drought tolerance by producing exopolysaccharides, ACC deaminase, and phytohormones; under salinity–alkalinity stress, plant growth-promoting rhizobacteria enhance salinity–alkalinity tolerance by regulating ion homeostasis and inducing host antioxidant systems [41,42]. For nutrient use efficiency, nitrogen-fixing bacteria, phosphate-solubilizing bacteria, and potassium-solubilizing bacteria convert soil nitrogen, phosphorus, and potassium into available forms that are otherwise inaccessible to plants, improving sorghum growth on infertile soils; arbuscular mycorrhizal fungi form symbiotic relationships with sorghum roots, expanding root absorption range and enhancing water and nutrient uptake efficiency [43]. Furthermore, the microbiome plays critical roles in sorghum ensiling and brewing processes: dynamic changes in lactic acid bacteria and other microorganisms during ensiling determine the fermentation quality of silage, while interactions between sorghum metabolites and microorganisms during Baijiu fermentation determine the formation of flavor compounds [44,45,46].

Co-improvement of the microbiome and host genome represents an important direction for future sorghum breeding. By treating “beneficial microorganism recruitment capacity” as a breeding target, combined with microbial inoculant technology, coordinated optimization of the host genome and microbiome can be achieved, developing green sorghum varieties with “low input, high output, and strong stress tolerance”.

## 3. Integrated Multi-Omics Precision Breeding Technology System in Sorghum

Multi-omics technologies have not only deepened understanding of the molecular mechanisms underlying traits, but also driven innovation in breeding technology systems, forming a precision breeding technology system centered on multi-omics data that substantially improves breeding efficiency and accuracy.

### 3.1. Multi-Omics Genomic Selection: Improving Prediction Accuracy for Complex Traits

Genomic selection (GS), which uses genome-wide markers to predict the genomic estimated breeding value of selection candidates, enabling early selection without full phenotypic evaluation. Conventional genomic selection primarily relies on SNP markers, exhibiting low prediction accuracy for complex traits such as yield and stress tolerance that are controlled by numerous minor-effect genes with strong genotype-by-environment interactions. Integration of multi-omics data provides new avenues to improve genomic selection accuracy: First, incorporating full-scale genetic variants including SVs, PAVs, and CNVs into prediction models compensates for variant effects untagged by SNP markers, improving coverage of genetic variation. Second, adding intermediate omics features such as transcriptome and metabolome as “endophenotypes” into models constructs hierarchical prediction models of “genotype—gene expression—metabolite—phenotype”, capturing effects of gene expression regulation on phenotypes. multi-layered regulatory network linking genotype, gene expression, metabolites and phenotype. Transcriptomic and metabolomic profiles are highly context-dependent across tissues, developmental stages and environments, an important caveat for multi-omics prediction models. Third, incorporating microbiome features into prediction models quantifies the contribution of the microbiome to phenotypes, improving prediction accuracy for varietal environmental adaptability. Fourth, adding epigenomic markers into models dissects phenotypic effects dependent on non-sequence variation, such as heterosis and stress memory [47,48,49]. In sorghum, multi-omics genomic selection achieves higher prediction accuracy than conventional SNP-only models, with published comparative studies reporting variable magnitudes of improvement depending on trait and population properties, including antioxidant content, biomass, and drought tolerance. As the cost of multi-omics data decreases, it will become a routine technology in sorghum breeding. Furthermore, integration of multi-omic markers with high-throughput phenotyping technologies further enhances breeding efficiency. For example, in drought tolerance breeding, molecular markers such as *SbNAC9* and *SbMYC2* correspond to seedling root architectural traits and root electrical capacitance—a classic physiological indicator of root cell viability under drought. Drone-based spectral imaging captures canopy-level spectral indices that reflect the overall activation of stress-responsive transcriptional and metabolic pathways. Together, they form a complete “molecular marker–physiological indicator–field phenotype” evaluation chain, enabling efficient early-generation screening for drought and other complex traits.

### 3.2. Multi-Omics-Guided Precision Editing: Enabling Targeted Modification of Breeding Targets

Genome editing technology is the core tool for achieving targeted trait improvement, and multi-omics research provides precise targets and editing strategy guidance for genome editing. In sorghum, CRISPR/Cas9 technology has been successfully applied to improve multiple traits: editing the α-kafirin gene family increased protein digestibility and lysine content in sorghum grains; editing the betaine aldehyde dehydrogenase gene *SbBADH2* created novel fragrant sorghum germplasm; editing strigolactone biosynthesis and transport genes developed sorghum germplasm resistant to Striga parasitism [50,51,52,53].

Multi-omics data guide the development of precision editing from three perspectives: First, target selection expands from coding regions to non-coding regulatory regions. Cis-regulatory elements identified through transcriptomics, epigenomics, and ATAC-seq data enable precise regulation of gene expression levels and patterns by editing promoter and enhancer regions, avoiding pleiotropic negative effects of complete gene knockout caused by coding region editing. Second, editing types expand from simple gene knockout to precise base editing and prime editing. Currently, adenine base editing (ABE) has been successfully applied in sorghum, creating herbicide-resistant *ALS* gene mutants; prime editing systems have also been established in sorghum, enabling precise base insertions, deletions, and substitutions [54,55]. Third, editing strategies expand from single-gene editing to multi-gene and pathway editing. Based on regulatory networks dissected by multi-omics, simultaneous editing of multiple key genes in pathways enables targeted improvement of complex traits; for example, editing multiple *MSD* genes in the jasmonic acid pathway substantially increases grain number per panicle. Editing individual regulators or biosynthetic steps in the MSD cascade (e.g., targeting MSD1 or MSD2) restores pedicellate spikelet fertility and thereby increases grain number per panicle. This represents a proposed breeding strategy rather than a fully demonstrated application. Furthermore, the development of epigenetic editing technologies (e.g., dCas9 fused to histone modification enzymes or DNA methyltransferases) enables regulation of gene expression without altering DNA sequence, providing new tools for heterosis utilization and stress memory fixation.

### 3.3. AI + Multi-Omics Intelligent Design: Supporting Customized Variety Molecular Design

The combination of artificial intelligence (AI) technologies and multi-omics data is the core of achieving intelligent design breeding in crops. By integrating multi-dimensional data including genomics, transcriptomics, epigenomics, phenomics, microbiomics, and enviromics, deep learning and other AI algorithms can construct accurate genotype-to-phenotype prediction models, simulating phenotypic effects of different allelic combinations, thereby designing optimal genotypes meeting breeding objectives [56]. In sorghum, AI + multi-omics has been applied in multiple scenarios: computer vision combined with high-throughput phenomics data enables automated and accurate identification of field sorghum plant height, biomass, panicle traits, and stress responses; machine learning models integrating multi-omics data predict gene functions and regulatory networks, substantially accelerating functional gene mining; genomic prediction models predict heterosis performance of different parental combinations, guiding selection of strong heterosis hybrids [50,57,58]. In the future, advances in AI technologies including large language models and multimodal models will enable the transition from “experience-based breeding” to “blueprint-based” intelligent design breeding, rapidly designing and developing customized sorghum varieties according to the needs of different ecological regions and end uses.

## 4. Applications of Multi-Omics Breeding for Core Agronomic Traits in Sorghum

Multi-omics technologies ultimately serve breeding practice. Focusing on the four core sorghum breeding objectives—stress tolerance, yield, quality, and nutrient use efficiency—multi-omics technologies have achieved a series of important breeding applications.

### 4.1. Stress Tolerance Traits: Multi-Layered Improvement of Abiotic and Biotic Stress Resistance

The core advantage of sorghum is its stress tolerance. Multi-omics technologies have dissected stress tolerance mechanisms from multiple layers, including host genomics, transcriptional regulation, and microbial interactions, driving precise development of stress-tolerant varieties. For abiotic stresses, drought tolerance is the most important stress tolerance trait in sorghum. Multi-omics studies have revealed multi-layered mechanisms of sorghum drought tolerance—at the genomic level, multiple key drought tolerance genes including *SbNAC9*, *SbWRKY30*, and *SbMYC2* have been identified, which improve drought tolerance by regulating ROS scavenging, ABA biosynthesis, and root development; at the transcriptional level, sorghum activates a large number of stress-responsive genes through the MAPK cascade and JA/ABA hormone pathways under drought stress, while regulating stress memory through epigenetic modifications; at the microbiome level, high-yielding genotypes actively recruit beneficial endophytes under drought, improving drought tolerance through microbe–host interactions [59,60,61,62]. For salinity–alkalinity tolerance, the major-effect tolerance gene *AT1* was cloned through GWAS; it encodes an atypical Gγ subunit and affects ROS homeostasis by regulating *PIP2;1* phosphorylation. This mechanism operates specifically under alkaline stress through aquaporin-mediated ion export. Knocking out this gene can increase sorghum yield by over 20% in saline–alkali soils, and this gene has conserved functions in rice, maize, wheat, and other crops, demonstrating great breeding application value [5]. For heat tolerance, integrated transcriptomic, metabolomic, and physiological multi-omics analysis revealed thermotolerance mechanisms regulated by heat shock proteins, raffinose biosynthesis, ROS scavenging, and the *SnRK1* pathway, identifying multiple heat tolerance breeding targets and providing genetic resources for addressing high temperature stress under climate warming [29]. Molecular pathways of drought tolerance directly correlate with quantifiable physiological indicators, which can be efficiently measured by high-throughput phenotyping platforms, forming a complete evaluation chain from molecular markers to physiological readouts and field phenotypes for efficient early-generation screening.

For biotic stresses, multi-omics studies have dissected sorghum resistance mechanisms against diseases, insect pests, and parasitic weeds: Dual RNA-seq technology dissected the interaction mechanism between sorghum and *Colletotrichum sublineola*, cloning the broad-spectrum antifungal gene *ARG1*, whose expression is regulated by histone modifications; high expression of *ARG1* significantly improves resistance to multiple fungal diseases [35]. Multiple resistance loci against aphids and stem borers were identified through GWAS, and transcriptomic analysis revealed insect resistance mechanisms of defensive metabolites such as tannins and cyanogenic glycosides [63]. Through pan-genome and structural variation analysis, the striga resistance gene *LGS1* and strigolactone transporter genes *SbSLT1*/*SbSLT2* were cloned; knocking out these genes through genome editing can reduce *Striga parasitism* rates by 67–94% without affecting sorghum growth, providing technical solutions for sorghum production in regions with severe Striga damage such as Africa [23,64].

### 4.2. Yield Traits: Systematic Optimization of Plant Architecture, Panicle-Grain Structure, and Photosynthetic Efficiency

Yield is the core objective of breeding. Sorghum yield is co-determined by multiple factors including plant architecture, grain number per panicle, grain weight, and photosynthetic efficiency. Multi-omics technologies have systematically dissected the genetic basis of these yield components, driving precise improvement of yield traits. For plant architecture, plant height is a key trait affecting lodging resistance and planting density. Currently, dwarfing genes including *Dw1*, *Dw2*, *Dw3*, and *Dw7a* have been cloned; they are involved in brassinosteroid signaling, auxin transport, and lipid signaling pathways, respectively, determining plant height by regulating internode cell elongation. Multi-omics studies have also revealed the mechanisms of plant height phenotypic plasticity, providing references for developing varieties with stable plant height across environments [65,66,67]. Flowering time is a key trait determining varietal ecological adaptability and yield potential. Six maturity genes *Ma1*-*Ma6* constitute the core regulatory network of photoperiodic flowering in sorghum. Natural mutations in these genes reduce photoperiod sensitivity, enabling sorghum to spread from its tropical origin to temperate regions; pyramiding these photoperiod-insensitive alleles allows development of maturity varieties adapted to different ecological regions [68,69,70]. For panicle-grain structure, genes including *MSD1*, *MSD2*, and *MSD3* determine fertility of pedicellate spikelets through regulating the jasmonic acid biosynthesis pathway; knocking out these genes can double grain number per panicle. G protein γ subunit genes such as *qTGW1a* and *SbDEP1* regulate grain weight and grain shape; pyramiding favorable alleles of these genes enables coordinated improvement of grain number and grain weight [71,72,73]. Furthermore, high-throughput phenomics technologies combined with UAVs and ground-based phenotyping platforms enable high-throughput and accurate identification of field yield-related traits, substantially improving selection efficiency for yield traits.

### 4.3. Quality Traits: Targeted Quality Regulation for End-Use-Specific Varieties

Sorghum has diversified end uses, with substantially different quality requirements for different applications. Multi-omics technologies provide technical support for targeted quality regulation of different end-use-specific sorghum varieties. Brewing sorghum is the dominant use of sorghum in China, requiring high tannin content, high amylopectin content, and appropriate protein content. Through metabolomics and molecular network analysis, key metabolites associated with Baijiu flavor in brewing sorghum were identified, revealing the molecular basis of flavor differences among sorghum varieties and providing quality evaluation indicators for brewing sorghum breeding [45]. Tannins are key quality traits for brewing sorghum. *Tan1* (WD40 protein) and *Tan2* (bHLH transcription factor) regulate condensed tannin biosynthesis by forming the MBW complex, while *Tan1* also affects bird feeding behavior by repressing biosynthesis of fatty acid-derived volatiles. Depending on end use, different *Tan1* alleles can be selected to develop high-tannin brewing varieties with bird resistance or low-tannin food/feed varieties [21,74]. Forage sorghum requires low tannin content, high protein digestibility, and low cyanogenic glycoside content. CRISPR editing of the kafirin gene family significantly improved sorghum protein digestibility and lysine content, solving the problem of low sorghum protein digestibility; key genes for cyanogenic glycoside biosynthesis were identified through metabolomic analysis, providing targets for developing low-cyanide forage sorghum [50,75]. Silage sorghum quality is closely associated with microbial dynamics and carbohydrate degradation during ensiling. Multi-omics studies revealed effects of different pretreatments (enzyme treatment, extrusion puffing) on microbial communities and carbohydrate fractions during ensiling, providing references for silage sorghum breeding and processing technology optimization [44,46]. These findings define key heritable determinants of silage quality: soluble sugar content and cell wall fiber composition are directly regulated by the sorghum genome, whereas pH decline rate and lactic acid bacteria colonization efficiency are composite host–microbe traits shaped by both plant genotype and environmental microbial communities. The associated QTLs and candidate genes provide direct targets for molecular breeding of silage sorghum, which should be pursued in conjunction with optimization of post-harvest processing and microbial conditions. Sweet sorghum and bioenergy sorghum require high stem sugar content, high biomass, and efficient deconstruction and conversion efficiency. The *Dry* gene encodes a NAC transcription factor, which is the key gene controlling stem juiciness; loss-of-function mutations cause parenchyma cell degradation and sugar accumulation in stems. Multi-omics studies have also revealed regulatory networks of stem secondary cell wall biosynthesis, as well as effects of drought stress on biomass and microbial conversion efficiency, providing targets for bioenergy sorghum improvement [76,77]. For lignocellulosic bioenergy applications, cell wall recalcitrance is a core trait determining biomass deconstruction and conversion efficiency. Multi-omics studies have shown that abiotic stresses such as drought remodel secondary cell wall composition—including lignin content, hemicellulose structure and cellulose crystallinity—by regulating cell wall biosynthetic and modifying genes, which in turn alters saccharification efficiency. These stress-associated cell wall regulators identified by omics provide targeted loci for breeding bioenergy sorghum with both high biomass yield and optimized deconstructability [78].

### 4.4. Nutrient Use Efficiency Traits: Green Variety Development Through Host–Microbiota Coordination

Excessive fertilizer application is a major source of agricultural non-point source pollution, and developing nutrient use efficient varieties is key to achieving green agriculture. Multi-omics technologies have dissected sorghum nutrient use efficiency mechanisms from two perspectives: host nutrient absorption and utilization, and microbial nutrient mobilization. For low nitrogen tolerance, integrated carbon–nitrogen metabolomic and transcriptomic analysis of sorghum germplasms with contrasting low nitrogen tolerance identified key metabolic pathways and responsive genes under low nitrogen stress, revealing regulatory mechanisms of carbon–nitrogen balance under low nitrogen conditions; nitrogen responses of sorghum CRISPR mutants were characterized using high-throughput phenotyping platforms, identifying multiple key nitrogen use efficiency genes and providing targets for nitrogen-efficient variety development [79,80]. For phosphorus use efficiency, sorghum adapts to low phosphorus soils through mechanisms including regulating root architecture, secreting organic acids, and recruiting phosphate-solubilizing microorganisms; transcriptomic studies revealed hormone signaling and phosphate transporter regulatory networks under low phosphorus stress. The microbiome plays critical roles in nutrient use efficiency: rhizosphere nitrogen-fixing bacteria, phosphate-solubilizing bacteria, and mycorrhizal fungi can significantly improve sorghum nitrogen and phosphorus uptake efficiency. Improving sorghum recruitment capacity for beneficial nutrient-mobilizing microorganisms through breeding, combined with microbial inoculants, can achieve the goal of green production with reduced fertilizer application without yield loss [81].

## 5. Challenges and Perspectives

Despite significant progress in sorghum multi-omics research and breeding applications, a series of bottlenecks remain: First, cross-omics data integration lacks unified standards and a systematic framework. The term multi-omics, as used herein, encompasses joint analysis of two or more omics layers, which has been widely conducted. However, higher-order systematic network integration—that is, dissection of complete regulatory networks spanning genomics, transcriptomics, epigenomics, and microbiomics—remains scarce. Data dimensions and noise levels vary substantially across different omics technologies, and efficient multi-omics data integration algorithms and standardized analytical pipelines are currently lacking. Most studies remain at the stage of single-omics analysis or simple two-omics correlation, and have not yet achieved true multi-layered regulatory network dissection. Second, to address the bottleneck of low genetic stability of epigenetic modifications and microbiome traits, technological breakthroughs are required. For epigenetics, epigenetic editing technologies can be applied to directionally fix favorable epigenetic marks, while screening for meiotically heritable epigenetic loci will improve the breeding utility of epigenetic traits. For the microbiome, systematic mapping of host mbQTLs that regulate microbiome assembly will identify core genotypes that stably recruit beneficial microorganisms. Combined with standardized inoculant technology, this will enable co-breeding of the host genome and microbiome. Third, sorghum genetic transformation efficiency remains low, with strong genotype dependence; most elite inbred lines are recalcitrant to genetic transformation, severely restricting functional gene validation and genome editing breeding applications. Fourth, multi-omics breeding costs remain relatively high; the costs of high-throughput sequencing and phenotyping remain prohibitive for conventional breeding programs, limiting widespread adoption of multi-omics technologies.

Future sorghum multi-omics research will expand toward higher resolution, more dimensions, and closer alignment with breeding applications: First, application of spatial multi-omics and single-cell multi-omics technologies will enable simultaneous profiling of transcriptome, epigenome, and chromatin state at single-cell resolution, systematically dissecting molecular mechanisms of cell differentiation and stress responses, and solving key biological questions such as C4 photosynthesis and vascular bundle development. Second, construction of graph-based pan-genomes will integrate genetic variation from all germplasms into a graph-based reference genome, completely solving alignment bias issues of single reference genomes and enabling accurate identification and utilization of full-scale genetic variation. Third, dissection of molecular mechanisms underlying host–microbiome interactions will map host genetic loci regulating microbiome composition (i.e., microbiome QTLs, mbQTLs) through GWAS, revealing molecular mechanisms by which hosts regulate microbiome recruitment, and enabling co-breeding of host genomes and microbiomes. Fourth, integration of synthetic biology and multi-omics will enable artificial design of high-value metabolic pathways (e.g., biosynthesis of special nutritional components and medicinal compounds) based on metabolic pathways and regulatory networks dissected by multi-omics, developing novel functional sorghum germplasms.

Future sorghum breeding will enter the era of multi-omics-driven intelligent design breeding, with the core being the construction of a complete technology system of “multi-omics data—AI models—breeding decisions”: First, construct a multi-omics database covering global sorghum germplasms, integrating multi-dimensional data including genomics, transcriptomics, epigenomics, phenomics, microbiomics, and enviromics, forming the “big data foundation” for sorghum breeding. Second, develop large model-based genotype-to-phenotype prediction models to achieve accurate cross-environment, cross-generation phenotype prediction, providing precise guidance for parental selection and progeny selection in breeding. Third, break through bottlenecks in efficient genetic transformation and large-scale genome editing technologies, achieving genotype-independent efficient transformation and multi-gene, multi-target precision editing, providing efficient operational tools for design breeding. Fourth, provided that breakthroughs in genetic transformation efficiency are achieved and the genetic basis of target traits is well understood, the breeding cycle for targeted improvement of elite varieties is expected to shorten from 8 to 10 years (conventional phenotypic breeding) to 2–3 years, supported by speed breeding technologies, early prediction via multi-omic genomic selection, and AI-driven targeted genotype design. This will support rapid development of customized sorghum varieties adapted to different ecological regions and industrial demands. For traits with complex genetic architecture and poorly understood mechanisms, longer cycles of multi-environment field validation remain necessary to provide core germplasm support for ensuring food security, developing marginal agriculture, and supporting the diversified sorghum industry. In rice, maize, and wheat, multi-omic genomic selection, precision genome editing guided by omics data, and AI-assisted parental design have been widely applied to improve yield, stress tolerance, and quality traits. These proven technical paths provide a clear reference for the construction of an intelligent breeding system in sorghum, but implementation must proceed incrementally in light of the unique characteristics of sorghum’s genetic transformation system.

Overall, current multi-omics breeding in sorghum remains focused on molecular target dissection. Elite germplasm development, multi-environment field validation, and industrial deployment represent weak points in the translational chain. Going forward, a full-process breeding pipeline of “intelligent design—rapid generation advancement—precision evaluation” should be established to bridge the gap between laboratory discoveries and field application, and to fully realize the complete breeding cycle of “mechanistic dissection–target mining–germplasm creation–field validation” depicted in Figure 2.

## Figures and Tables

**Figure 1:**
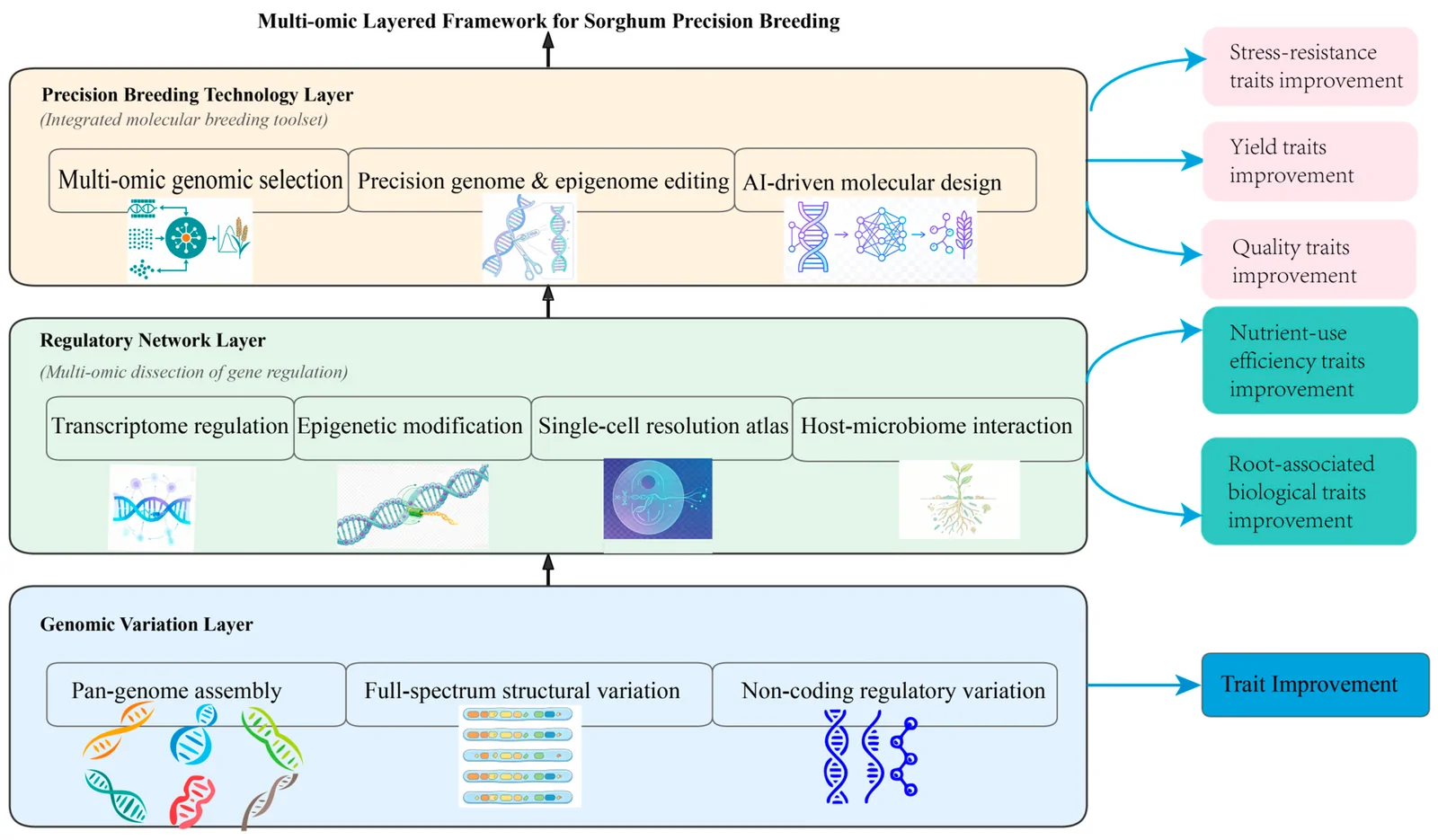
Multi-omic layered framework for sorghum precision breeding. This framework is hierarchically organized into three interconnected layers spanning from genetic basis dissection to regulatory mechanism elucidation and breeding application translation, forming a complete research chain linking genotype to phenotype. The bottom Genomic Variation Layer captures the full spectrum of intraspecific genetic diversity through pan-genome assembly, genome-wide structural variation identification, and non-coding regulatory variation mining, providing the fundamental genetic basis for trait dissection. The middle Regulatory Network Layer bridges the genotype–phenotype gap by dissecting trait formation mechanisms at multiple resolutions, including transcriptome dynamics, epigenetic modifications, single-cell-resolution gene expression atlases, and host–microbiome interactions. The top Precision Breeding Technology Layer translates multi-omic discoveries into applied breeding tools, comprising multi-omic genomic selection, precision genome and epigenome editing, and AI-driven molecular design. These integrated technologies collectively enable targeted improvement of five core sorghum trait categories: stress-resistance traits, yield traits, quality traits, nutrient use efficiency traits, and root-associated biological traits, ultimately supporting the development of customized, high-performance sorghum varieties.

**Figure 2:**
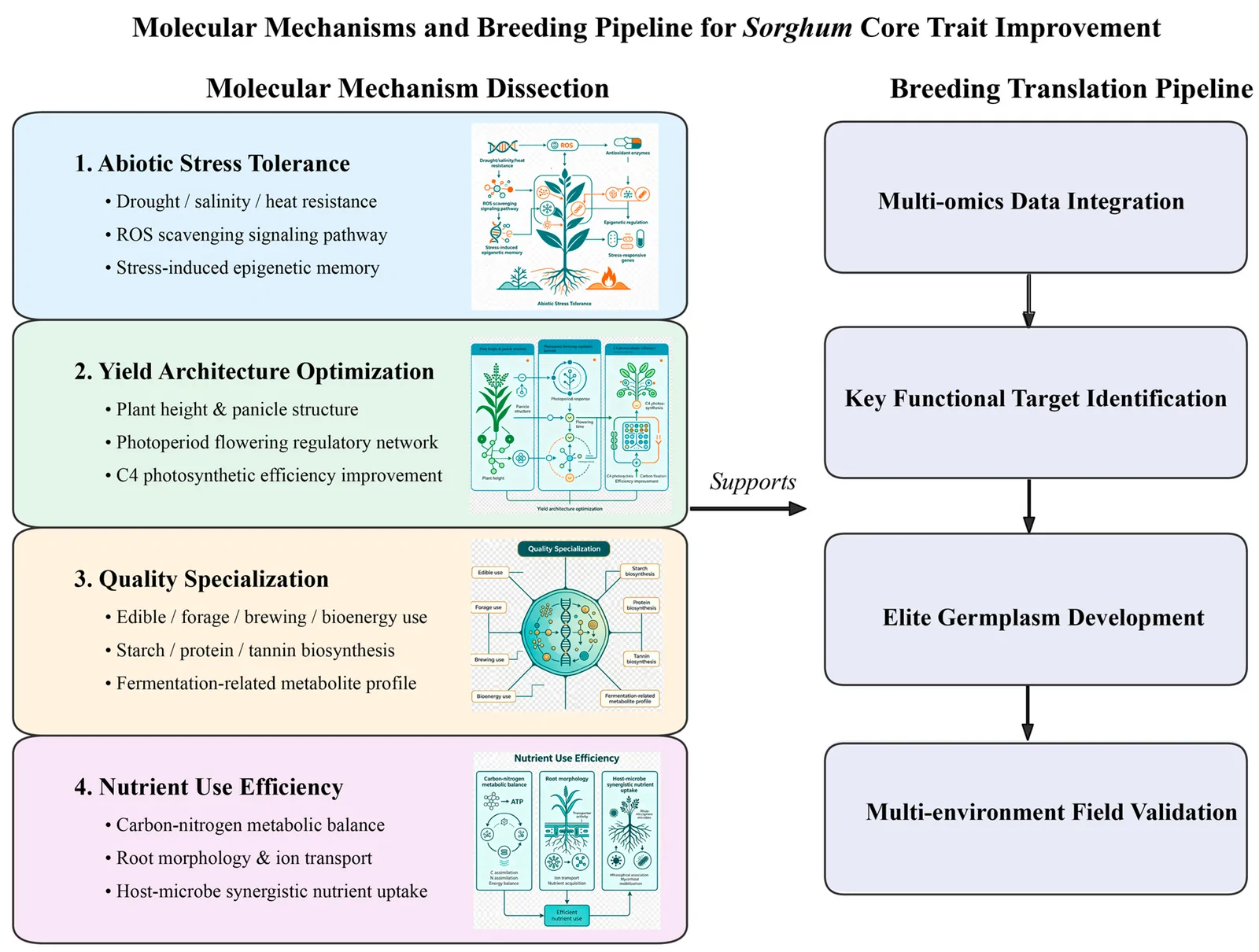
Molecular mechanisms and breeding pipeline for sorghum core trait improvement. This figure illustrates the translational framework connecting multi-omic molecular mechanism dissection to applied sorghum breeding practice. The left panel systematically summarizes the molecular regulatory mechanisms underlying four core sorghum breeding objectives: (1) Abiotic stress tolerance, conferred by drought/salinity/heat resistance pathways, reactive oxygen species (ROS) scavenging signaling cascades, and stress-induced epigenetic memory; (2) Yield architecture optimization, determined by plant height and panicle structure regulation, photoperiod-dependent flowering regulatory networks, and C4 photosynthetic efficiency improvement; (3) Quality specialization for diverse end uses (edible, forage, brewing, and bioenergy), regulated by starch, protein, and tannin biosynthesis pathways as well as fermentation-related metabolite profiles; (4) Nutrient use efficiency, mediated by carbon–nitrogen metabolic balance, root morphology and ion transport regulation, and host–microbe synergistic nutrient uptake. The right panel depicts the stepwise breeding translation pipeline, proceeding from multi-omics data integration, key functional target identification, and elite germplasm development to multi-environment field validation. Mechanistic insights from the left panel directly support the precision and efficiency of this pipeline, enabling targeted development of sorghum varieties with optimized agronomic traits and stable performance across environments.

## Data Availability

No new data were created or analyzed in this study. Data sharing is not applicable to this article.
